# *Talaromyces atroroseus*, a New Species Efficiently Producing Industrially Relevant Red Pigments

**DOI:** 10.1371/journal.pone.0084102

**Published:** 2013-12-19

**Authors:** Jens C. Frisvad, Neriman Yilmaz, Ulf Thrane, Kasper Bøwig Rasmussen, Jos Houbraken, Robert A. Samson

**Affiliations:** 1 Department of Systems Biology, Technical University of Denmark, Kongens Lyngby, Denmark; 2 CBS–KNAW Fungal Biodiversity Centre, Utrecht, The Netherlands; 3 Department of Biology, Utrecht University, Utrecht, The Netherlands; Pacific Northwest National Laboratory, United States of America

## Abstract

Some species of *Talaromyces* secrete large amounts of red pigments. Literature has linked this character to species such as *Talaromyces purpurogenus, T. albobiverticillius, T. marneffei*, and *T. minioluteus* often under earlier *Penicillium* names. Isolates identified as *T. purpurogenus* have been reported to be interesting industrially and they can produce extracellular enzymes and red pigments, but they can also produce mycotoxins such as rubratoxin A and B and luteoskyrin. Production of mycotoxins limits the use of isolates of a particular species in biotechnology. *Talaromyces atroroseus* sp. nov., described in this study, produces the azaphilone biosynthetic families mitorubrins and *Monascus* pigments without any production of mycotoxins. Within the red pigment producing clade, *T. atroroseus* resolved in a distinct clade separate from all the other species in multigene phylogenies (ITS, β-tubulin and *RPB1*), which confirm its unique nature. *Talaromyces atroroseus* resembles *T. purpurogenus* and *T. albobiverticillius* in producing red diffusible pigments, but differs from the latter two species by the production of glauconic acid, purpuride and ZG–1494α and by the dull to dark green, thick walled ellipsoidal conidia produced. The type strain of *Talaromyces atroroseus* is CBS 133442

## Introduction

*Monascus* species are known to produce six major azaphilone pigments being the yellow monascin and ankaflavin; the orange monascorubrin and rubropunctatin and the red monascorubramine and rubropunctamine, in addition to more than 20 related pigments [1,2]. Another azaphilone series of yellow pigments is even more widespread in *Talaromyces*, i.e. the mitorubrins [3–5]. The red pigment producer *Monascus purpureus* has been used primarily in Southern China, Japan and Southeast Asia for making red rice wine, red soybean cheese and Anka (red rice) [6]. A problem is that some samples of *Monascus*–fermented rice have been found to contain the mycotoxin citrinin [7], but also that *Monascus* isolates also often produce mevinolin, a drug that is also unwanted in foods [2]. The production of such mycotoxins and drugs limits the use of *Monascus* for industrial purposes, but since citrinin has not been found in any *Talaromyces* species, the latter may be a good alternative for red pigment production. Studies have shown that polyketide azaphilone *Monascus* red pigments and/or their amino acid derivatives are naturally produced by *Talaromyces aculeatus*, *T. pinophilus*, *T. purpurogenus* and *T. funiculosus* [8,9]. *Talaromyces amestolkiae*, *T. ruber* and *T. stollii* also produce azaphilone polyketides, as recently described by Yilmaz et al. [10], but in those three species the pigment are not diffusing into the growth medium. *Talaromyces amestolkiae* and *T. stollii* were isolated from immuno-compromised patients and are potential human pathogens, while *T. purpurogenus* produces mycotoxins such as rubratoxins A and B, rugulovasins, and luteoskyrin [10]. These factors limit the use of these species for biotechnological production of azaphilone pigments.

In the current study we describe a new *Talaromyces* species, *T. atroroseus*, which secretes large amounts of *Monascus* red pigments, without the production of any known mycotoxins.

## Materials and Methods

### Strains

Cultures were obtained from the CBS-KNAW Fungal Biodiversity Centre culture collection, Utrecht, the Netherlands. Fresh isolates deposited in the working collection of the Department of Applied and Industrial Mycology (DTO) housed at CBS, and strains from the IBT collection at DTU Systems Biology in Kongens Lyngby, Denmark were also included in this study. Strains are listed in Table 1. KAS strain numbers are from the fungal collection of Keith A. Seifert, Ottawa, Canada.

**Table 1:** Strains used in this study of *Talaromyces atroroseus* and related species.

**CBS No.**	**Other Collection No.**	**Species**	**Information and Origin**
206.89	IFO 6580, IBT 3960; DTO 41F4	*T. albobiverticillius*	Unknown, Japan
238.95	IBT 11181, CBS 123796	*T. atroroseus*	Red sweet bell pepper, Kgs. Lyngby, Denmark
234.60	DTO 37A4	*T. atroroseus*	Unknown, Germany
257.37	DTO 37A3	*T. atroroseus*	Ex air in nitrite factory, Germany
313.63	DTO 41G2	*T. albobiverticillius*	*Vitis vinifera* fruit, South Africa
364.48	ATCC 9777, IMI 040037, NRRL 1061,	*T. atroroseus*	Unknown, Darien, Manchuria, China
	QM 6760, DTO 178A3, IBT 4458, IBT 11180		
391.96	DTO 41G8	*T. atroroseus*	Unknown, Tanzania
113139	IBT 3967, NRRL 1147, DTO 177I2	*T. atroroseus*	Unknown, USA
113167	DTO 39I2, DTO 39I3	*T. albobiverticillius*	Unknown, unknown
113168	IBT 31347, DTO 39H9, DTO 177I9	*T. albobiverticillius*	Sputum of patient, male, Copenhagen,
			Denmark
113153	IBT 3458, NRRL 1136, DTO 37A7	*T. atroroseus*	Ex mixed culture, Arlington Farm, Virginia
			USA
124294	IBT 23082	*T. atroroseus*	Tropical rainforest, Peru
133440	BCRC 34774, DTO 166E5, IBT 31667	Type of *T. albobiverticillius*	Decaying leaves of a broad–leaved tree, Taiwan
133441	BCRC 34775, DTO 166E6, IBT 31668	*T. albobiverticillius*	Decaying leaves of a broad–leaved tree. Taiwan
133442	KAS 3778, DTO 178A4, IBT 32470	Type of *T. atroroseus*	House dust, South Africa
133443	IBT 29388, DTO 189D4	*T. atroroseus*	Contamination in petri dish, Lyngby, Denmark
133444	IMI 163167, IBT 23702, DTO 189C2	*T. albobiverticillius*	*Punica granata*, unknown
133447	DTO 81I2	*T. atroroseus*	Swab sample from cheese warehouse,
			the Netherlands
133448	DTO 157G5	*T. albobiverticillius*	Pomegranate, Turkey
133449	IBT 29464, DTO 189D5	*T. atroroseus*	Mouse dung, Høve Strand, Denmark
133450	FRR 75, IBT 4454, DTO 188I9	*T. atroroseus*	Soil Murrumbidgee irrigation Area,
			New South Wales, Australia
133452	NRRL 2120, IBT 3547, DTO 193H9	*T. albobiverticillius*	Cotton duck, Panama
113154	R.B., IMI 090178, NRRL 1214, IBT 3645,	*T. atroroseus*	”Parasite” in *Aspergillus niger* culture,
	IBT 4428, CBS 127571		Kansas City, Missouri, USA
	TA85S-28-H2, AZ ,IAM 15392, JCM 23216, IBT 32650	*T. atroroseus*	Soil, Thailand
	IBT 20955	*T. atroroseus*	Air root in white mangrove,
			Can de Aruca, Paria Bay, Venezuela
	IBT 4466	*T. albobiverticillius*	*Punica granata*, imported to Denmark

### Morphological analysis

Macroscopic characters were studied on agar media Czapek-Dox yeast autolysate agar (CYA), CYA supplemented with 5 % NaCl (CYAS), yeast extract sucrose agar (YES), creatine sucrose agar (CREA), dichloran 18 % glycerol agar (DG18), oatmeal agar (OA) and malt extract agar (Oxoid) (MEA). The isolates were also tested on CYA at 37 °C and on Blakeslee malt extract agar (MEA2). All media were prepared as described by Samson et al. [11]. The strains were inoculated in three points onto media in 90-mm Petri dishes and incubated for 7 d at 25 °C in darkness. After incubation, the colony diameters on the various agar media were measured. Colonies were photographed with a Canon EOS 400D. Species were characterized microscopically by preparing slides from MEA. Lactic acid was used as mounting fluid. Specimens were examined using a Zeiss AxioSkop2 plus microscope.

### DNA extraction, PCR amplification and sequencing

Strains were grown for 7 to 14 d on MEA prior to DNA extraction. DNA was extracted using the Ultraclean^TM^ Microbial DNA isolation Kit (MoBio, Solana Beach, U.S.A.). The extracted DNA was stored at -20 °C. The ITS regions, regions of the β-tubulin and *RPB1* genes were amplified and sequenced according to methods previously described [12–15].

### Data analysis

**Figure 1:**
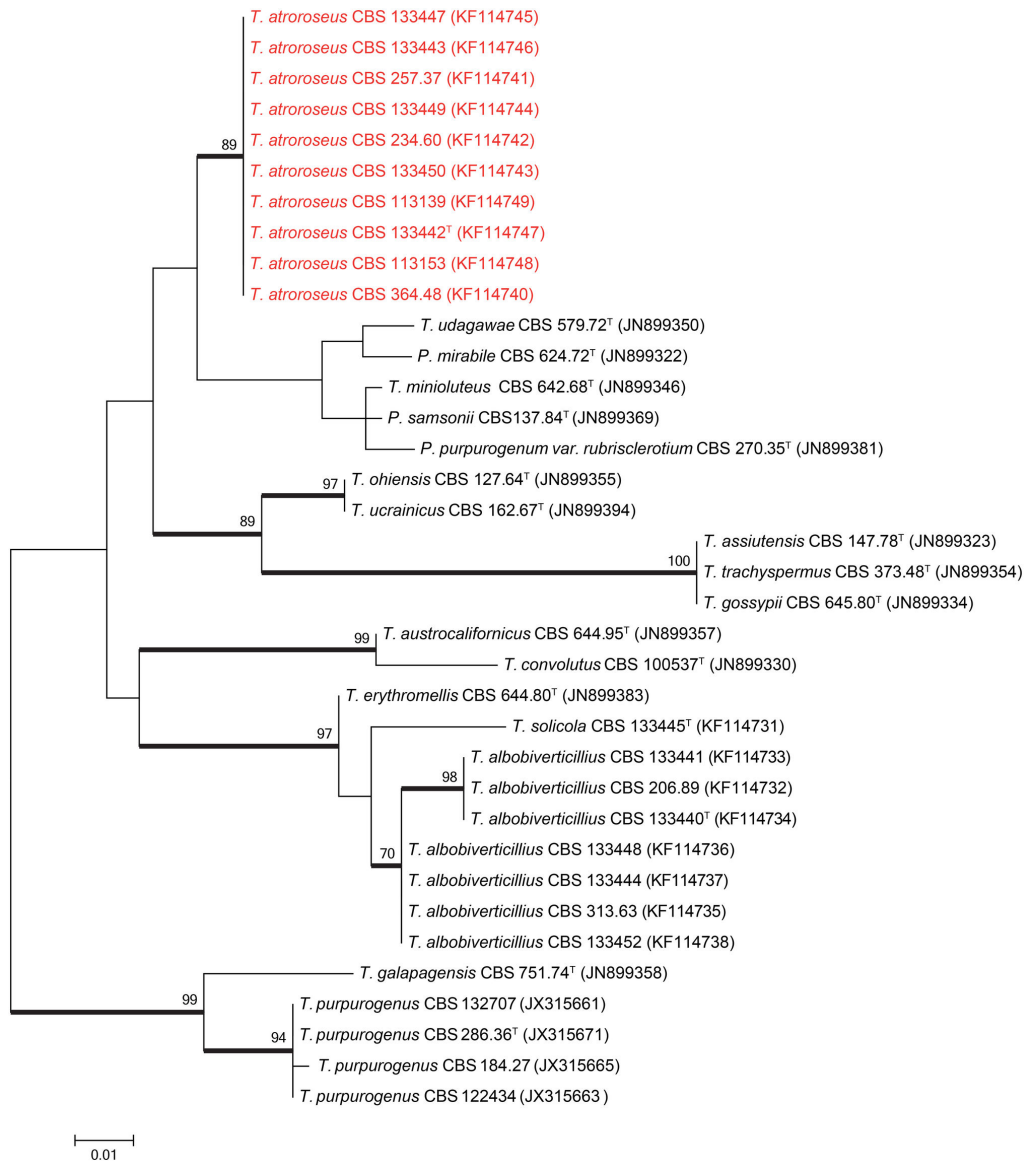
Maximum likelihood tree comparing the ITS gene region of *Talaromyces* species closely related to *T. atroroseus*. *Talaromyces galapagensis* and *T. purpurogenus* were used as outgroup. Support in nodes is indicated above thick branches and is represented by bootstrap values higher than 70%. GenBank accession numbers are given between brackets, (^T^ = ex-type). Red coloured names indicate *T. atroroseus* strains.

**Figure 2:**
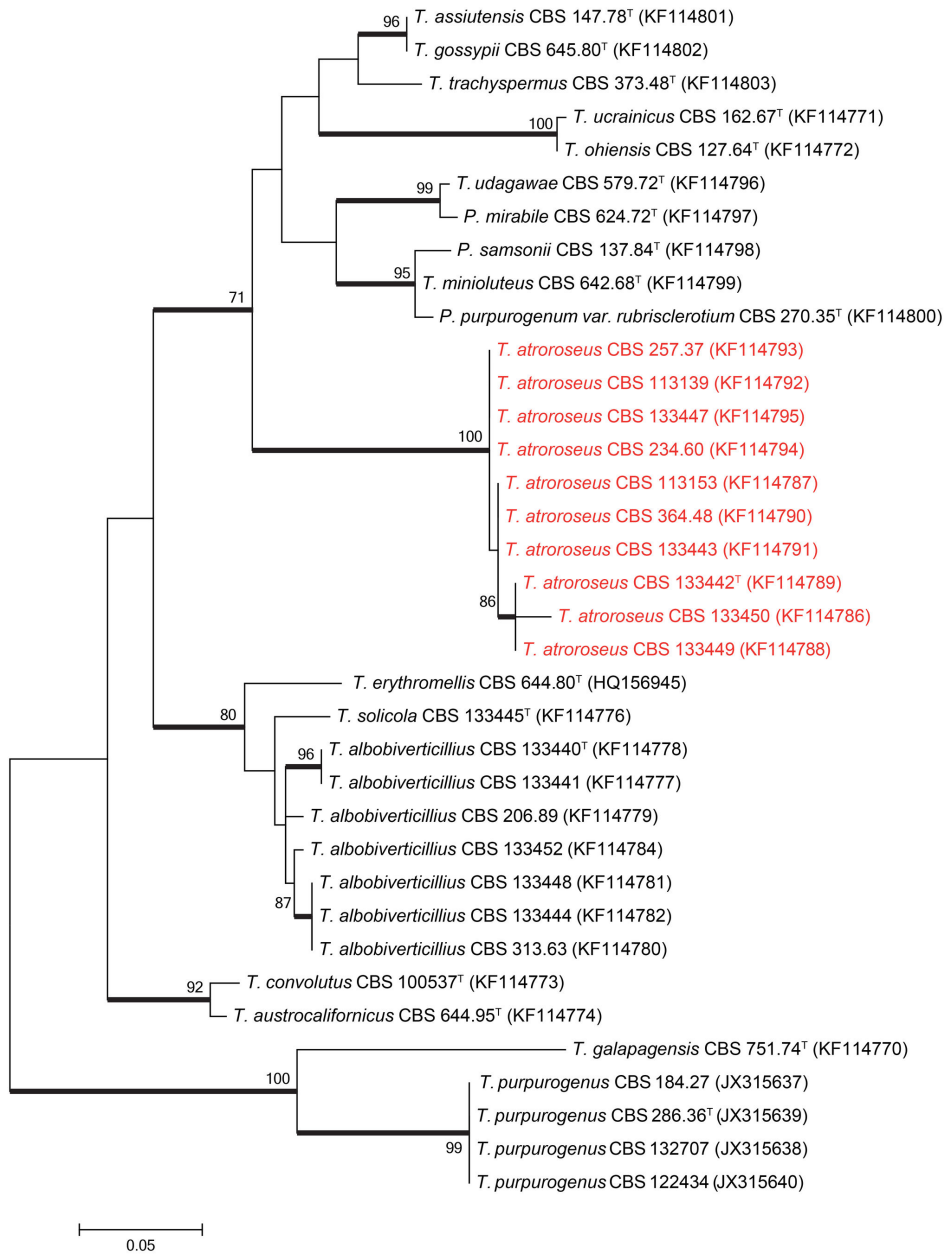
Maximum likelihood tree comparing the β–tubulin gene region of *Talaromyces* species closely related to *T. atroroseus*. *Talaromyces galapagensis* and *T. purpurogenus* were used as outgroup. Support in nodes is indicated above thick branches and is represented by bootstrap values higher than 70%. GenBank accession numbers are given between brackets, (^T^ = ex-type). Red coloured names indicate *T. atroroseus* strains.

**Figure 3:**
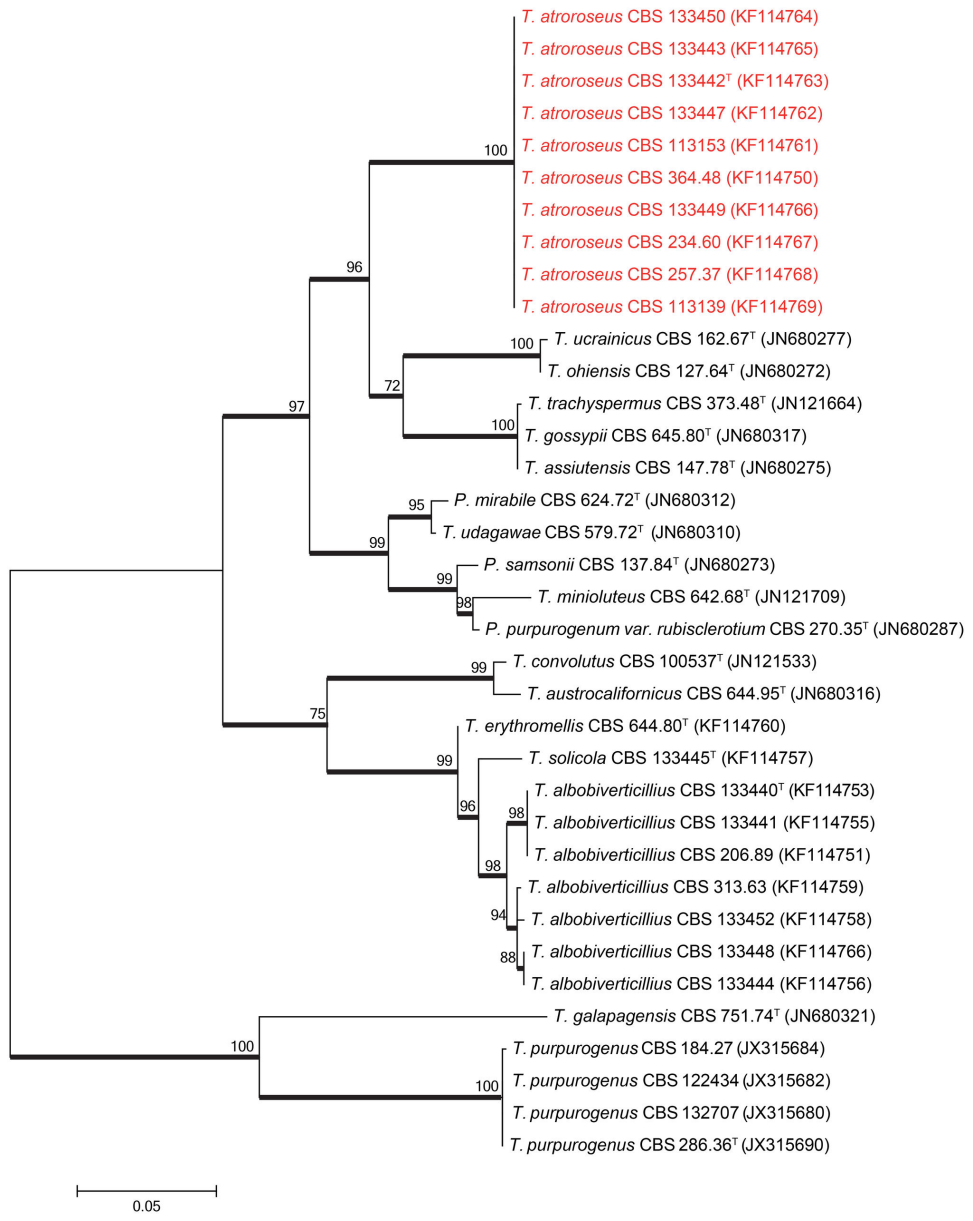
Maximum likelihood tree comparing the *RPB1* gene region of *Talaromyces* species closely related to *T. atroroseus*. *Talaromyces galapagensis* and *T. purpurogenus* were used as outgroup. Support in nodes is indicated above thick branches and is represented by bootstrap values higher than 70%. GenBank accession numbers are given between brackets, (^T^ = ex-type). Red coloured names indicate *T. atroroseus* strains.

Sequence contigs were assembled using Seqman from DNAStar Inc. Newly generated ITS, β-tubulin and *RPB1* sequences were included in a data set obtained from the Samson et al. [15] study. Data sets were aligned using Muscle software within MEGA5 [16]. Neighbour–joining analysis on the individual data sets was performed in MEGA5 and confidence in nodes determined using bootstrap analysis with 1000 replicates. *Talaromyces galapagensis* (CBS 751.74^T^) was selected as a suitable out-group in all the phylogenies. The newly generated sequences were deposited in GenBank (accession numbers, see Table 1 and Figures 1–3). 

### Extrolites

Cultures grown on CYA and YES for 7 d at 25 °C were used for extrolite extractions. Extracts were analysed by HPLC using alkylphenone retention indices and diode array UV–VIS detection as described by 17–19, using three 6 mm agar plugs. Standards of extrolites from the collection at DTU Systems Biology (Denmark) were used to compare the extrolites from the species under study [18].

The extrolite extractions from *T. atroroseus* CBS 133450, CBS 113154 and CBS 123796 were also analysed by ultra high performance liquid chromatography high-resolution mass spectrometry (UHPLC-HRMS). Liquid chromatography was performed on an Agilent 1290 Infinity LC system with a DAD-detector coupled to an Agilent 6550 iFunnel Q-TOF with an electrospray ionization source. The separation was performed on a 2.1 x 250 mm, 2.7 μm Poroshell 120 Phenyl-Hexyl column (Agilent) at 60 °C with a water-acetonitrile gradient (both with 20 mM formic acid) going from 10 % (vol/vol) to 100 % acetonitrile in 15 min followed by 2.5 min with 100 % acetonitrile and then returning to the start conditions for 2.5 min for equilibration before next sample. All time the flow rate was kept at 0.35 mL/min. HRMS was performed in ESI^+^ and extrolites were identified with targeted search on accurate mass of [M+H]^+^ and [M+Na]^+^ using Agilent MassHunter Qualitative Analysis B.06.00 software and a database of potential extrolites in *T. atroroseus* with support from UV-VIS spectra. The list of compounds searched for including the extrolite standards can be found in Table S1.

### Nomenclature

1. The electronic version of this article in Portable Document Format (PDF) in a work with an ISSN or ISBN will represent a published work according to the International Code of Nomenclature of algae, fungi, and plants, and hence the new names contained in the electronic publication of a PLOS ONE article are effectively published under that Code from the electronic edition alone, so there is no longer any need to provide printed copies. In addition, new names contained in this work have been submitted to MycoBank from where they will be made available to the Global Name Index. The unique MycoBank number can be resolved and the associated information viewed through any standard web browser by appending the MycoBank number contained in this publication to the prefix http://www.mycobank.org/MB. The online version of this work is archived and available from the following digital repositories. PubMed Central, LOCKSS.
2. Repository of *Talaromyces atroroseus* Yilmaz, Frisvad, Houbraken & Samson 2013 sp. nov. [urn:lsid:mycobank.org: 804901]

## Results and Discussion

The relationship between the *Talaromyces atroroseus* sp. nov. and its close relatives were studied using multigene phylogenies, bason on ITS, *RPB1* and β-tubulin sequences. The aligned datasets were 482, 888 and 374 bp long, respectively. The new species resolved in a clade together with other red pigment producing species such as *T. albobiverticillius*, and *T. minioluteus*. *Talaromyces purpurogenus* resolved in a distantly related clade (Figures 1–3). Within the red pigment producing clade, *T. atroroseus* resolved in a distinct clade separate from all the other species in all three phylogenies, confirming its unique nature.

Historically red pigment production caused a lot of confusion and resulted in numerous misidentifications in literature. This is especially true for *Talaromyces purpurogenus*, *T. ruber*, *Penicillium sanguineum* and *P. crateriforme*. *Penicillium purpurogenum* and *P. rubrum* were described by Stoll [20]. In their monograph Raper and Thom [21] also described *P. purpurogenum* and *P. rubrum*. No type material was available for *P. rubrum* therefore Raper and Thom [21] used two strains to describe *P. rubrum*, NRRL 1062 (= CBS 370.48) and NRRL 2120 (= CBS 133452). Pitt [22] synonymized *P. rubrum*, *P. crateriforme* and *P. sanguineum* with *P. purpurogenum*. The issues in the *T. purpurogenus* complex were clarified by Yilmaz et al. [10] who synonymized *Penicillium crateriforme* and *P. sanguineum* with *T. purpurogenus* and they described *T. ruber* as a distinct species. NRRL 1062 remained as *T. ruber* but NRRL 2120 (= CBS 133452) is a different species than *T. ruber*. Our results showed that NRRL 2120 is *T. albobiverticillius*. Raper and Thom [21] based the *Penicillium purpurogenum* description on NRRL 1061 (= CBS 364.48). However our results show that NRRL 1061 is a typical *T. atroroseus* strain.

**Figure 4:**
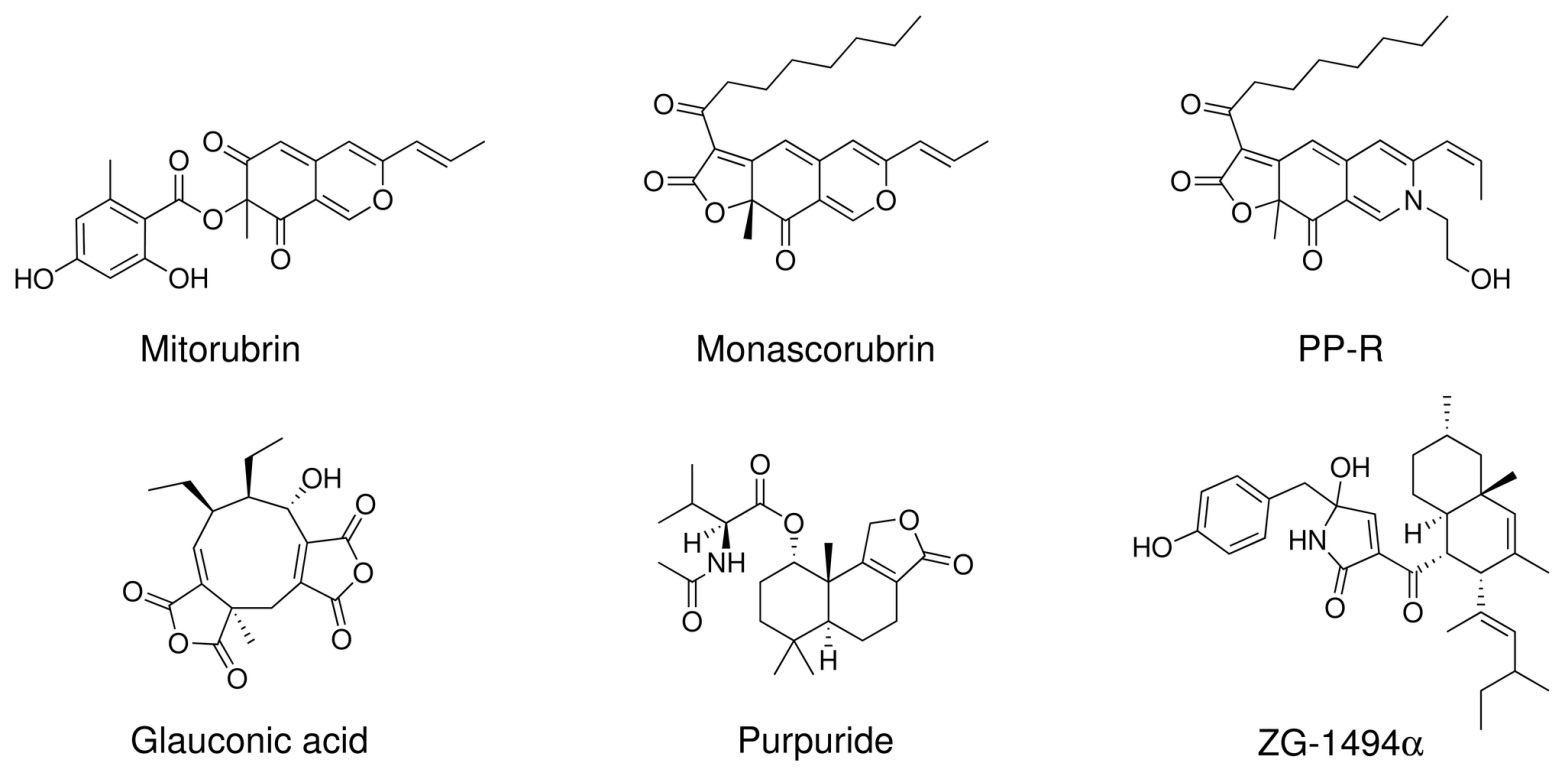
Structures of some of the most characteristic compounds produced by *Talaromyces atroroseus*. All six compounds were detected in this study.

Both *Talaromyces purpurogenus* and *T. atroroseus* are common in soil, indoor environments, and fruits. *Talaromyces atroroseus* resembles *T. purpurogenus* and *T. albobiverticillius* in producing red diffusible pigments, but differs from the latter two species by the production of glauconic acid, purpuride and ZG–1494α (Table 2 and Figure 4) and by the dull to dark green thick walled ellipsoidal conidia produced. Barton et al. [26,27] and Barton and Sutherland [28] reported glauconic acid from *P. purpurogenum* IMI 090178, which in the present study has been re-identified as *T. atroroseus*, while ZG–1494α was reported from *P. rubrum* CBS 238.95 [36], which is also a typical *T. atroroseus*. *Talaromyces atroroseus*, *T. purpurogenus* and *T. albobiverticillius* differ from *T. ruber*, *T. amestolkiae* and *T. stollii* by their production of red diffusible pigment. In Table 3 many red pigment producers identified as *Penicillium* species are listed, that may either be *T. purpurogenus, T. ruber, T. albobiverticillius* or *T. atroroseus*. The strains listed in Table 3 were not available for us, so their exact identity cannot be verified.

**Table 2:** Reported extrolite production by strains verified as *Talaromyces atroroseus* during this study.

**Extrolite**	**Reported producer**	**Culture collection numbers**	**Reference**
Glaucanic acid, Glauconic acid	*Penicillium “R. B.”, P. purpurogenum*	**R.B.** = IMI 090178 = NRRL 1214 = CBS 113154 = IBT 3645 = IBT 4428	[23–30]
N-glutarylmonascorubramine, N-glutarylrubropunctamine	*P. purpurogenum*	**IBT 11181** = CBS 238.95 = CBS 123796	[9]
N-glutarylmonascorubramine	*P. purpurogenum*	R.B. = IMI 090178 = NRRL 1214 = CBS 113154 = **IBT 3645** = IBT 4428	[9]
Monascorubramine, PP-R	*P. purpurogenum*	**IBT 11180** = CBS 364.48 = ATCC 9777 = IMI 040037 = NRRL 1061 = QM 6760 = IBT 4458	[9]
PP-V, PP-R, PP-O, PP-Y	P. sp.	TA85S-28-H2 = **AZ** = IAM 15392 = JCM 23216 = IBT 32650	[43–47]
Purpuride	*P. purpurogenum*	**CBS 257.37**	[31]
Purpurogenone, Deoxypurpurogenone	*P. purpurogenum*	**CBS 257.37**	[32–35]
ZG-1494α	*P. rubrum*	IBT 11181 = **CBS 238.95** = CBS 123796	[36]

Strain numbers in bold are the strain numbers used in the references.

**Table 3:** Reported extrolite production from strains potentially belonging to *Talaromyces atroroseus*, but not examined during this study.

**Extrolite**	**Reported producer**	**Strain identifier / Culture collection number**	**Reference**
2,6,7-trihydroxy-3-methyl-naphthalene-1,4-dione	*Penicillium purpurogenum*	JS03-21*	[37]
BE-25327	*P. purpurogenum*	F25327 = FERM P-12345	[38]
Dhilirolide A, B, C, D	*P. purpurogenum*	IMI 357108	[39]
Glauconic acid	*P. glaucum*	-*	[40]
Gluconic acid	*P. purpurogenum* var. *rubrisclerotium* (= *T. pinophilus*)	No. 2670 = NRRL 1064 = CBS 270.35 = ATCC 4713 = ATCC 52224 = NRRL 1142 = IBT 4302	[41]
(-)-Mitorubrin,	*P. purpurogenum*	JS03-21*	[37]
*Monascus* red pigment	P. sp.	HKUCC 8070	[42]
Orsellinic acid	*P. purpurogenum*	JS03-21*	[37]
Purpactin A, B, C	*P. purpurogenum*	FO-608 = FERM P-10776	[48,49]
Purpurester A, B	*P. purpurogenum*	JS03-21*	[37]
Purpurquinones A, B, C	*P. purpurogenum*	JS03-21*	[37]
Red W59 (C_30_H_34_O_9_N_3_)	*P. purpurogenum*	-*	[50]
Red pigment	*P. purpurogenum*	GH2*	[51–53]
Red pigment	*P. purpurogenum*	SX01*	[54]
Red pigments	*P. purpurogenum*	DPUA 1275	[56,57]
Red pigments	*P. purpurogenum*	-*	[58,59]
Red pigments	P. sp.	-*	[60]
SL 3238 (C_27_H_41_NO_7_)	*P. purpurogenum*	NRRL 3364	[55]
TAN-931	*P. purpurogenum*	JS03-21*	[37]

Based on the reported morphology and extrolites the strains in the table are by the authors’ judgement belonging to *Talaromyces atroroseus* or a closely related species.

^*^ Strain not deposited in any accessible culture collection

**Figure 5:**
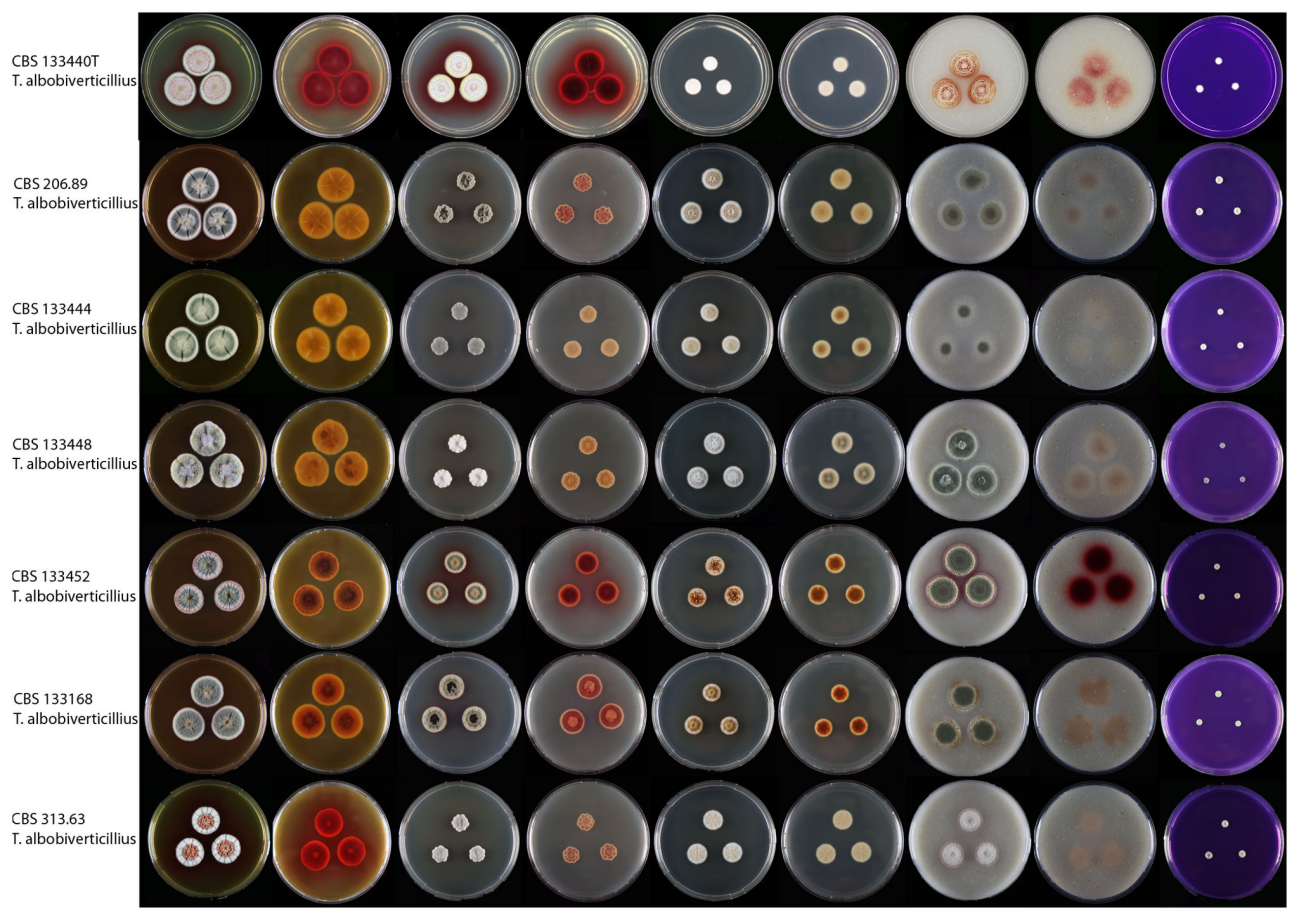
Strains of *Talaromyces albobiverticillius* on MEA, CYA, DG18, OA and CREA. Colony obverse and reverse is shown for the first four media and obverse for CREA.

Many *Talaromyces* species produce striking diffusing red pigments, especially *T. purpurogenus*, *T. atroroseus, T. albobiverticillius, T. minioluteus*, and *T. marneffei*. These red pigments are typically composed of the azaphilone pigments (Figure 5) monascorubrin, rubropunctatin, threonine derivative of rubropunctatin, monascorubramine, PP-R (= 7–(2–hydroxyethyl)-monascorubramine), rubropunctamine, N-glutarylrubropunctamine, and PP–V [8,9,43,44,61]. The same family of azaphilones are also known from red rice, where different species of *Monascus* have grown [1,2]. These red pigments are of interest for the industry as they are stable and non-toxic and can be used as food colorants [62]. The azaphilone pigments can react with amino acids, hence their name, and give intense dark red colours. In addition some of these species produce yellow azaphilone pigments, such as monascin, ankaflavin, monascusone A and B, xanthomonascin A, and another series of yellow mitrorubrin azaphilones: mitorubrin, mitorubrinol, mitorubrinol acetate, mitorubrinic acid, and many other related compounds [5]. Many of these pigments have been reported from or found in *T. atroroseus* in this study (Table 2 and Table 4). The potential for pigment production has in this study only been investigated in small scale on solid media; however, *T. atroroseus* also produce pigments in liquid cultures under the right conditions [8,46]. The potential for up scaling the production of red pigments needs to be investigated thoroughly.

**Table 4:** Extrolites of *Talaromyces atroroseus* and *T. albobiverticillius* as examined by HPLC-DAD and/or UHPLC- HRMS and comparison to standards on the media CYA and YES.

**Species**	**Culture collection number**	**Extrolites** * **found**
*T. atroroseus*	CBS 133450^a^	glauconic acid^b^, monascorubrin^b^, PP-R^b^, purpuride^b^, purpuroquinone A^b^, ZG-1494α^b^
	CBS 113154^a^	glauconic acid, N-glutarylmonascorubramine^b^, monascorubrin^b^, PP-O^b,^ PP-R^b^, purpuride^b^, purpuroquinone A^b^, ZG-1494α^b^
	CBS 123796^a^	FK17-P2b2^b^, glauconic acid, N-glutarylmonascorubramine^b^, mitorubrin, mitorubrinol, monascorubrin^b^, PP-O^b^, PP-R^b^, purpuride^b^, purpuroquinone A^b^, purpurogenone, ZG-1494α^b^
	CBS 257.37	monascorubramine, purpuride, several Monascus-red pigments
	CBS 234.60	glauconic acid, monascorubramine, purpuride, ZG-1494α
	CBS 391.96	glauconic acid, monascorubramine, purpuride, ZG-1494α
	CBS 364.48	glauconic acid, monascorubramine, PP-R, purpuride, rubropunctatin, ZG-1494α
	CBS 133447	Glauconic acid, purpuride
	CBS 133442	Glauconic acid, monascorubramin, purpuride, rubropunctatin
	CBS 113153	glauconic acid, mitorubrin, monascorubramine, monascorubrin, purpuride
	CBS 113139	monascin, monascorubramine
	IBT 3933	glauconic acid, mitorubrin, monascorubramin, a purpactin
	IBT 20955	glauconic acid, monascorubramine, monascorubrin, purpuride, ZG-1494α
	IBT 23082	PP-R (only tested for *Monascus* pigments)
	CBS 133443	glauconic acid, monascorubramine, purpuride
	CBS 133449	glauconic acid, monascorubrin, purpuride
	JCM 23216	Glauconic acid, monascorubramine, purpuride
*T. albobiverticillius*	CBS 113168	mitorubrin, mitorubrinic acid, monascorubramine, PP-R, rubropunctatin, vermicellin
	CBS 313.63	mitorubrin, monascorubramin, monascorubrin, rubropunctatin
	IBT 4466	mitorubrinic acid, monascorubramine, a purpactin
	CBS 113167	mitorubrin, mitorubrinic acid, monascorubrin, a purpactin
	CBS 133444	mitorubrin, mitorubrinic acid, mitorubrinol
	CBS 133452	mitorubrin, mitorubrinic acid, monascorubramine, rubropunctatin
	CBS 133441	mitorubrin, mitorubrinic acid, monascin, monascorubramin, rubropunctatin, vermicellin

^a^ Strains examined by both HPLC-DAD and UHPLC- -HRMS

^b^ Extrolites identified by UHPLC- -HRMS

^*^ The extrolites only identified by HPLC-DAD might in some cases not be the actual metabolite but a derivative with the same chromophore and retention on the column

**Figure 6:**
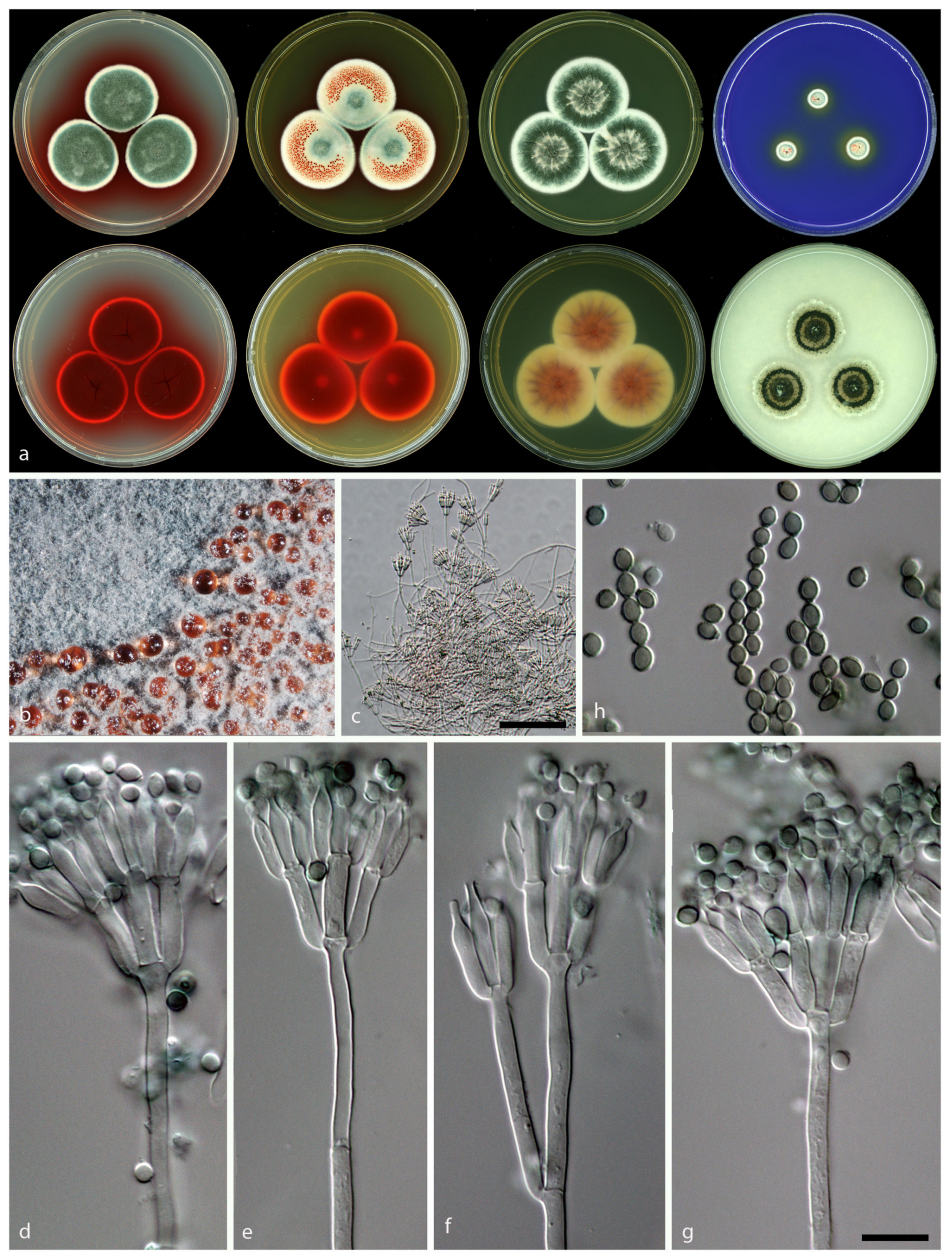
Morphological features of *Talaromyces atroroseus* sp. nov. CBS 133442. a: Colonies incubated on CYA, CYA reverse, MEA, MEA reverse, YES, YES reverse, CREA and OA from left to right b: Colony texture on MEA, c–g: Conidiophores produced on MEA; h: Conidia. (– Scale Bar in c = 50 µm, in g = 10 µm and applies to d–h).

 Even though sequence variations were observed for *Talaromyces albobiverticillius* strains, morphologically they were similar. Two strains used for the original description of *T. albobiverticillius* were received from Dr. Sung-Yuan Hsieh [63]. These included the type strain CBS 133440^T^ and CBS 133441. These strains were isolated from soil in Taiwan and produce white conidial masses and intense soluble red pigment on various media (Figure 6). However, other freshly isolated *T. albobiverticillius* strains produce densely sporulating colonies and do not show any stability for red pigment production. Some of the *Talaromyces albobiverticillius* strains did not produce any soluble pigment such as CBS 133444 and CBS 133448. Strains that did produce red pigments include CBS 113168, and CBS 133452. On MEA only the degraded or mutated strains of *T. albobiverticillius*, such as CBS 133440^T^ and CBS 313.63 produced red pigments. Micromorphologically all *T. albobiverticillius* strains produce long stipes (up to 380 µm) (Figure 5). Two strains of *T. albobiverticillius* (CBS 133440^T^ and CBS 133441) have globose to subglobose, smooth conidia; however, the remaining strains produce ellipsoid to fusiform smooth conidia (Figure 5).

Even though two clades were observed in the phylogenies there are no concordance between observed clades and morphological characters as discussed above. As such, they are considered here as representing one species. Raper and Thom [21] mentioned a number of colour mutations they observed in strains of *P. citrinum* and *P. chrysogenum*. They stated that colour mutations are encountered as the most common and conspicuous types of mutations, especially considering mature conidia. Mutations can often be observed when a strain loses its green pigment in its conidia, resulting in a white or tanned colour. Colour mutants are regularly encountered among the strains which were exposed to artificial stimulations such as ultra-violet, X-ray radiations and neutron bombardment [21].

*Talaromyces atroroseus* is considered as the optimal producer of industrially important yellow and red soluble pigments. Another option as a suitable producer of red soluble azaphilone pigments is *T. albobiverticillius*. However *T. albobiverticillius* produces soluble red pigment only in some strains. We speculate that the mitorubrins produced by *Talaromyces atroroseus* are of the (-)-form, as they have been shown to be that for the closely related *Talaromyces purpurogenus* (at that time identified as *Penicillium rubrum*) [64,65]. However, Natsume et al. [66] and Suzuki et al. [67] found both (+) and (-)-forms in the genus *Talaromyces*, while mitorubrins in *Hypoxylon* and other related genera are of the (+)-form [68–70]. Although *T. purpurogenus* is another good producer of diffusible red azaphilone pigments, this species also produce a series of mycotoxins, such as rubratoxin A and B and luteoskyrin in addition to extrolites that may be toxic if injected intraperitoneally (spiculisporic acid) [71] or in the veins of cats (rugulovasine A and B) [72,73]. *Talaromyces purpurogenus* can thus not be recommended for industrial production for red pigments.

*Talaromyces atroroseus* Yilmaz, Frisvad, Houbraken & Samson *sp. nov*. Figure 6.

Mycobank MB804901 [urn:lsid:mycobank.org: 804901]

**Holotype:** CBS 133442 in Centraalbureau voor Schimmelcultures is designated as the holotype of *Talaromyces atroroseus*. It was isolated from indoor house dust, Stellenbosch, South Africa by C. Visagie in 2010. 

**Cultures ex type:** CBS 133442 = IBT 32470 = DTO 178A4 = KAS 3778 

**Etymology:** Named after the dark rosy diffusing azaphilone pigment mixture produced.

**Diagnosis:** Dark green ellipsoidal rough-walled conidia and a dark red diffusing pigment, strains of the species produce the unique combination of secondary metabolites: glauconic acid, ZG–1494α, purpuride, red *Monascus* pigments, mitorubrins, and purpactins in fresh isolates.

**CYA 25 °C 7d:** Colonies are 30–40 mm in diameter, low, plane; margins narrow (1–2 mm), entire, low; mycelia white; texture velvety; sporulation dense, conidia *en masse* dark to dull green; exudate absent; soluble pigment red; reverse coloration dark cherry red.

**MEA 25 °C 7d:** Colonies 35–40 mm in diameter, low, plane, having a pinkish colour because of exudates diffusing into mycelia; margins narrow (1–2 mm), entire, low; mycelia white; texture velvety overlaying floccose; sporulation moderately dense, conidia *en masse* bluish green; exudate red droplets especially close to margin; soluble pigment absent, after prolonged incubation red pigments produced; reverse coloration dark red.

**YES 25 °C 7d:** Colonies are 33–45 mm in diameter, raised at centre, sulcate; margins wide (2–3 mm), entire, low; mycelia white; texture velvety; sporulation dense, conidia *en masse* dark to dull green; exudates small red droplets; soluble pigment red in some isolates; reverse coloration brownish red.

**CYAS 25 °C 7d:** Commonly no growth, some strains up to 5 mm in colony diameter.

**CREA 25 °C 7d:** Colonies 9–13 mm in diameter, weak acid production close to colony periphery, some strains acid absent; reverse dark red.

**OA 25 °C 7d:** Colonies 30–35 mm in diameter, low, plane; margins wide (2–3 mm), entire, low; white mycelia; texture velvety; sporulation dense; conidia *en masse* dull to dark green, almost appears blackish green; exudates absent; soluble pigment absent; reverse coloration commonly greenish yellow to green, red in some isolates.

**DG18 25 °C 7d:** Colonies 27–30 mm in diameter, low, plane; margins wide (2 mm), entire, low; mycelia white; texture velvety, floccose mycelia present at centre; sporulation dense, conidia *en masse* greyish green, at margins bluish green; exudates absent; soluble pigment absent; reverse colour is beige.

Conidiophores mostly biverticillate, subterminal branches produced, have a greenish to brownish pigmentation; Stipes smooth walled, 90–150 × 2.5–3 µm; Branches 2–3 when present, 15–50 × 2–3 µm; Metulae in verticils of 3 to 5 per stipe, 8–15 × 3.0–4.0 µm; Phialides acerose, 3 to 6 per metula, 9.5–12.5 × 2.5–3 µm; Conidia rough walled, ellipsoidal, 2–3.5 × 1.5–2.5 µm.

**Figure 7:**
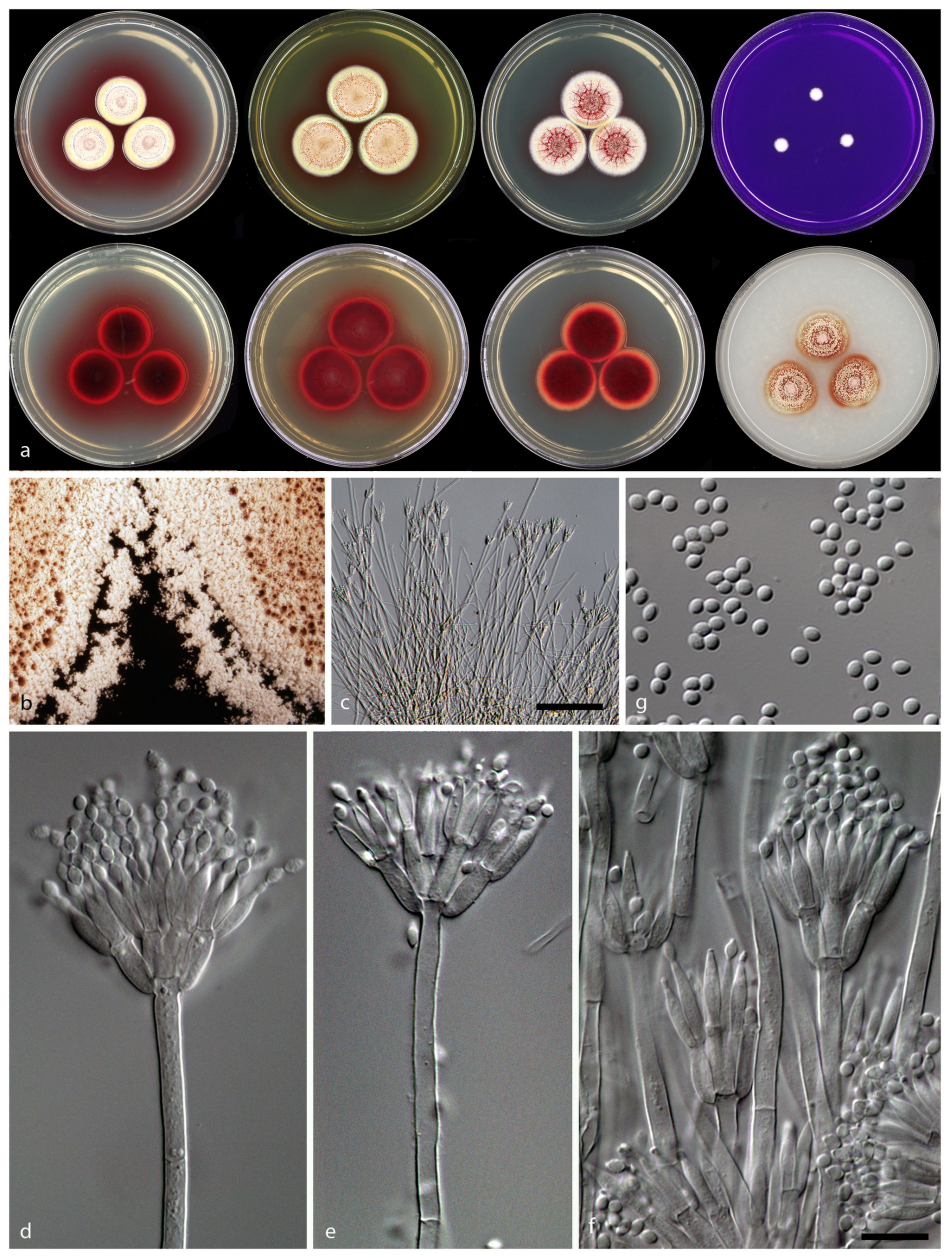
Morphological features of *Talaromyces albobiverticillius* CBS 133440. : Colonies incubated on CYA, CYA reverse, MEA, MEA reverse, YES, YES reverse, CREA and OA from left to right b: Colony texture on MEA, c–f: Conidiophores produced on MEA; g: Conidia. ( – Scale Bar in c = 50 µm, in f = 10 µm and applies to d–g).

***Talaromyces albobiverticillius*** (H.–M. Hsieh, Y.–M. Ju & S.–Y. Hsieh) Samson, Yilmaz, Frisvad & Seifert, Studies in Mycology 70: 174, 2011. MycoBank MB560683 (Figure 7)

**Type. BCRC 34774**

**CYA 25 °C 7d:** Colonies 15–20 mm in diameter, low, crateriform, in some isolates sulcate; margins narrow (1–2 mm), entire, low; mycelia white and yellow; texture floccose to velvety; sporulation sparse, in some isolates moderately dense; conidia *en masse* when sparse white, otherwise greyish green; exudates red small droplets; soluble pigmentation red; reverse coloration dark cherry red.

**MEA 25 °C 7d:** Colonies 24–28 mm in diameter, low, crateriform, in some isolates sulcate; margins wide (2–3 mm), entire, low; mycelia white and yellow; texture velvety with overlaying floccose in the centre; sporulation sparse, in some isolates moderately dense; conidia *en masse* when sparse white, otherwise greyish green; exudates clear and red droplets; soluble red pigment absent; reverse coloration dark red.

**YES 25 °C 7d:** Colonies 23–25 mm in diameter, raised at centre, sulcate; margins wide (2–3 mm), entire, low; mycelia white and yellow; texture velvety; sporulation sparse, in some isolates moderately dense; conidia *en masse* when sparse white, otherwise greyish green; exudates small orange to red droplets; soluble pigment red in some strains; reverse coloration red to pale brown.

**CYAS 25 °C 7d:** No growth.

**CREA 25 °C 7d:** Colonies 4–8 mm in diameter, no acid produced.

**OA 25 °C 7d:** Colonies 25–28 mm in diameter, low, plane; margins wide (3–4 mm), entire, low; mycelia white; texture velvety; sporulation sparse to moderately dense; conidia *en masse* when sparse white, otherwise greyish green; exudates absent; soluble pigment absent; reverse coloration red in the centre and the rest greenish yellow to green.

**DG18 25 °C 7d:** Colonies 15–35 mm in diameter, low, plane; margins narrow (1–2 mm), entire, low; mycelia white; texture velvety; sporulation sparse; sparse to moderately dense; conidia *en masse* when sparse white, otherwise greyish green; exudates clear to red droplets; soluble pigment red in some isolates absent; reverse coloration brownish red, in some isolates beige.

Conidiophores strictly biverticillate, subterminal branches absent; stipes smooth walled, 200–380 × 2.5–3.5 µm; metulae in verticals of 3–6, 8–12 × 1.5–4.5 μm; phialides acerose, 3–7 per metula, 8–13.5 × 2–3 μm; conidia smooth to finely roughened, spheroid to subglobose, in some isolates fusiform, 2–3.5 (4) × 1.5–2.5 μm.

## Conclusion

*Talaromyces atroroseus* is a new species that produce large amounts of red pigments that can be potentially used for colouring foods, as it does not produce any known mycotoxins. Certain strains of *T. albobiverticillius* may also be used for these purposes.

## Supporting Information

Table S1 **Table S1 contains the extrolites searched for by ultra high performance-liquid chromatography-diode array detection-high resolution mass spectrometric detection (UHPLC-DAD-HRMS) the fungal extracts analysed**. The table also includes data on the available standards used in the study.
(DOCX)
