# Supplementary material for: Talaromyces atroroseus, a New Species Efficiently Producing Industrially Relevant Red Pigments
Source: PLoS One. 2013 Dec 19;8(12):e84102. doi: 10.1371/journal.pone.0084102 (PMC3868618; doi:10.1371/journal.pone.0084102)
Supplement: Table S1 — contains the extrolites searched for by ultra high performance-liquid chromatography-diode array detection-high resolution mass spectrometric detection (UHPLC-DAD-HRMS) the fungal extracts analysed. The table also includes data on the available standards used in the study. (DOCX) [file pone.0084102.s001.docx]

Supplementary material.

*Talaromyces atroroseus*, a new species efficiently producing industrially relevant red pigments.

By

Jens C. Frisvad, Neriman, Yilmaz, Ulf Thrane, Kasper Bøwig Rasmussen, Jos Houbraken, Robert A. Samson

Supplementary Table 1 (**Table S1**)

**Table S1:** Library of compounds searched for by UHPLC-DAD-HRMS including available standards

| Compound | Formula | Monoisotopic Mass | Adducts/Losses observed, ESI^+^ | RT | Confirmed by UV/VIS | Standard Available |
| --- | --- | --- | --- | --- | --- | --- |
| Ankaflavin | C23H30O5 | 386.2093 | [M+H]^+^=387.2169,  [M+Na]^+^=409.1988 | 12.418 |  | Yes |
| Deoxypurpurogenone | C29H20O10 | 528.1056 | [M+H]^+^=529.1134 | 11.299 | Yes | No |
| Dhilirolide A | C25H28O9 | 472.1733 |  |  |  | No |
| Dhilirolide B | C25H28O8 | 456.1784 |  |  |  | No |
| Dhilirolide C | C25H28O8 | 456.1784 |  |  |  | No |
| Dhilirolide D | C25H30O7 | 442.1992 |  |  |  | No |
| FK17-P2b2 | C13H16O4 | 236.1049 | [M+H]^+^=237.1130, [M+Na]^+^=259.0952,  [M-H_2_O+H]^+^= 219.1026 | 6.150 |  | No |
| Glauconic acid | C18H20O7 | 348.1209 | [M+H]^+^=349.1284, [M+Na]^+^=371.1105,  [M+NH_4_]^+^=366.1551, [M-H_2_O+H]^+^= 331.1176 | 7.991 / 8.846 |  | Yes |
| (+)-Mitorubrin | C21H18O7 | 382.1053 | [M+H]^+^=383.1127, [M+Na]^+^=405.0951,  [M-H_2_O+H]^+^= 365.1024 | 9.478 | Yes | Yes |
| (+)-Mitorubrinic acid | C21H16O9 | 412.0794 | [M+H]^+^=413.0873, [M+Na]^+^=435.0687,  [M-H_2_O+H]^+^= 395.0763 | 7.198 | Yes | Yes |
| (+)-Mitorubrinol | C21H18O8 | 398.1002 | [M+H]^+^=399.1079, [M+Na]^+^=421.0894,  [M-H_2_O+H]^+^= 281.0964 | 7.126 | Yes | Yes |
| (+)-Mitorubrinol acetate | C23H20O9 | 440.1107 | [M+H]^+^=441.1187, [M+Na]^+^=463.1003 | 8.846 | Yes | Yes |
| Monaphilone A | C22H32O4 | 360.2301 |  |  |  | No |
| Monaphilone B | C20H28O4 | 332.1988 |  |  |  | No |
| Monaphilone C | C20H32O4 | 336.2301 |  |  |  | No |
| Monascin | C21H26O5 | 358.1780 | [M+H]^+^=359.1851,  [M+Na]^+^=381.1666 | 11.438 | Yes | Yes |
| Monascorubramine | C23H27NO4 | 381.1940 | [M+H]^+^=382.2015, [M+Na]^+^=404.1836 | 10.307 | Yes | Yes |
| Monascorubrin | C23H26O5 | 382.1780 | [M+H]^+^=383.1856, [M+Na]^+^=405.1675 | 12.528 | Yes | Yes |
| Monascusone A | C13H18O5 | 254.1154 |  |  |  | No |
| Monascusone B | C17H18O5 | 302.1154 |  |  |  | No |
| Monascuspurpurone | C21H30O5 | 362.2093 |  |  |  | No |
| Monasfluore A | C21H24O5 | 356.1624 |  |  |  | No |
| Monasfluore B | C23H28O5 | 384.1937 |  |  |  | No |
| N-glutarylmonascorubramine | C28H33NO8 | 511.2206 | [M+H]^+^=512.2279, [M+Na]^+^=534.2100 | 9.521 | Yes | No |
| N-glutarylrubropunctamine | C26H29NO8 | 483.1893 |  |  |  | No |
| PP-O | C23H24O7 | 412.1522 | [M+H]^+^=413.1598, [M+Na]^+^=435.1415 | 10.761 | Yes | No |
| PP-R | C_25_H_31_NO_5_ | 425.2202 | [M+H]^+^=426.2279, [M+Na]^+^=448.2096 | 10.050 | Yes | No |
| PP-V | C23H25NO6 | 411.1682 |  |  |  |  |
| Purpactin A | C23H26O7 | 414.1679 |  |  |  | No |
| Purpactin B | C23H26O7 | 414.1679 |  |  |  | No |
| Purpactin C | C23H24O7 | 412.1522 |  |  |  | No |
| Purpurester A | C13H16O5 | 252.0998 |  |  |  | No |
| Purpurester B | C12H12O4 | 220.0736 |  |  |  | No |
| Purpuride | C22H33NO5 | 391.2359 | [M+H]^+^=392.2429, [M+NH_4_]^+^= 409.2696, [M+Na]^+^=414.2256 | 9.110 | Yes | No |
| Purpurogenone | C29H20O11 | 544.1006 | [M+H]^+^=545.1079 | 11.106 | Yes | Yes |
| Purpurquinone A | C21H20O9 | 416.1107 | [M+H]^+^=417.1186, [M+Na]^+^=439.1004 | 7.383 | Yes | No |
| Purpurquinone B | C21H20O8 | 400.1158 |  |  |  | No |
| Purpurquinone C | C21H20O10 | 432.1056 |  |  |  | No |
| Rubratoxin A | C26H32O11 | 520.1945 |  |  |  | Yes |
| Rubratoxin B | C26H30O11 | 518.1788 |  |  |  | Yes |
| Rubropunctamine | C21H23NO4 | 353.1627 | [M+H]^+^=354.1704 [M+Na]^+^=376.1523 | 8.961 | Yes | Yes |
| Rubropunctatin | C21H22O5 | 354.1467 |  | 11.489 |  | Yes |
| Rugulovasine A | C16H16N2O2 | 268.1212 |  |  |  | No |
| Rugulovasine B | C16H16N2O2 | 268.1212 |  |  |  | Yes |
| Xanthomonasin A | C21H24O7 | 388.1522 |  |  |  | No |
| Xanthomonasin B | C23H28O7 | 416.1835 |  |  |  | No |
| ZG-1494a | C32H43NO4 | 505.3192 | [M+H]^+^=506.3267, [M+Na]^+^=528.3093,  [M-H_2_O+H]^+^= 488.3161 | 12.009 |  | No |

The table contains the used for targeting search on accurate mass. The observed adducts and losses confirming the identity of a compound have been reported for standards as well as identified compound in this study. Furthermore it is stated where UV/VIS spectra have been used in the confirmation of compound identity.

RT: Retention time relating to the UHPLC method described.
